# Unveiling Cell‐Type‐Specific Immune Reactions in Human Blood Following Varied Fruit and Vegetable Blends Interventions

**DOI:** 10.1002/mnfr.70238

**Published:** 2025-09-01

**Authors:** Yueqin He, Julia N. DeBenedictis, Simone G. van Breda, Theo M. de Kok

**Affiliations:** ^1^ Department of Translational Genomics GROW – Research Institute For Oncology and Reproduction Maastricht University Maastricht the Netherlands

**Keywords:** cell‐type‐specific analysis, chronic disease prevention, CIBERSORTx, fruits and vegetables (F&Vs), immune cell responses

## Abstract

A diet rich in fruits and vegetables (F&Vs) reduces chronic disease risk by modulating oxidative stress, inflammatory cytokines, and immune cell activity in the blood. Given the complexity of peripheral blood and its cellular components, understanding cell‐type‐specific responses to F&V interventions remains essential and challenging. We used CIBERSORTx to analyze immune cell fractions and gene expression profiles from RNA sequencing data of the MiBLEND study, which assessed the impact of seven F&V blends on chronic disease markers, phytochemical absorption, and gene expression changes in blood. Analysis of white blood cells (WBCs) showed no significant changes in proportions of major leukocyte types. CIBERSORTx determined proportions of 22 leukocyte subtypes, correlating with traditional WBC composition. Paired *t* tests revealed that blends higher in carotenoids, anthocyanins, or complex phytochemicals altered CD4+/CD8+ T cell ratios. A strong positive association was observed between the proportion of memory B cells and beta‐carotene levels in peripheral blood following the consumption of Blend 1. Finally, DEGs and pathway analyses showed that some blends affected DNA repair in B cells, NF‐kappa B signaling in plasma cells, and endocytosis in dendritic cells (DCs) and natural killer (NK) cells. These findings offer clearer insight into immune cell dynamics in blood following F&V interventions, clarifying immune mechanisms involved in their preventive effects.

## Graphical abstract

Paired peripheral‐blood RNA‐seq from MiBLEND was deconvolved with CIBERSORTx to profile leukocyte composition and cell‐type‐specific transcriptomic responses to seven fruit‐and‐vegetable blends. Deconvolution yielded 22 leukocyte subtypes that correlated with laboratory WBC reports (*R* = 0.886, *p* ≤ 0.01). Blends 2, 3, and 7 shifted T‐cell composition, notably CD8 T cells and resting memory CD4 T cells. Group‐mode profiles enabled differential‐expression and KEGG analyses that identified cell‐type‐specific pathways: DNA repair in B cells, NF‐κB signaling in plasma cells, and endocytosis in mast cells, dendritic cells, and NK cells, including N‐glycan biosynthesis. This approach provides mechanistic insight into cell‐type‐specific immune responses associated with fruit‐and‐vegetable intake.

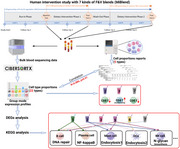

## 1 Introduction

A diet rich in fruits and vegetables (F&Vs) is strongly recommended for its potential to provide protection against a range of chronic diseases like cancers and cardiovascular disease [1, 2, 3, 4]. This protective effect is thought to be mediated among others by the regulation of oxidative stress and the immune system by a variety of antioxidants and immune‐protective phytochemicals, including flavonoids, anthocyanins, carotenoids, and glucosinolates [5, 6]. Key indicators include IL‐6, TNF‐α, and C‐reactive protein, as well as oxidative stress markers like 8‐hydroxy‐2′‐deoxyguanosine (8‐OHdG), malondialdehyde (MDA), and reactive oxygen species (ROS) in peripheral blood were usually found changed following dietary intervention [7, 8]. It is also important to note that the peripheral blood comprises multiple layers and subsets of cells, each equipped with distinct and complementary functions. This interconnected cellular network operates in a harmonized manner to protect the host from a wide range of stimuli, including infections and the development of cancer, as well as various physiological interactions [9]. Consequently, it is crucial, but challenging, to comprehend the behavior of all different types of immune cells following a dietary intervention with F&Vs.

CIBERSORTx is an analytical tool that can be used to generate an estimation of the abundances of member cell types in a mixed cell population, using bulk gene expression data without physical cell isolation [10, 11]. In this study, we employed it on the RNA sequencing data from the MiBLEND study [12] to investigate the specific changes in immune cell fractions and transcription of genes. The MiBLEND study is a randomized crossover study in which the participants were randomly assigned to consume different blends of F&Vs. Each blend represented a major phytochemical class (flavonoids, anthocyanins, carotenoids, and glucosinolates) or combinations thereof. The MiBLEND study aimed to investigate changes in ex vivo‐induced DNA damage (%tail DNA, tail moment, and 8‐OHdG), oxidative stress levels (TEAC, superoxide), retinal microvasculature (CRAE, CRVE, AVR, branching, and tortuosity, as a marker of cardiovascular health), phytochemical absorption, and gene expression in whole blood. The results demonstrate that increasing F&V intake from low to recommended levels significantly elevated plasma antioxidant capacity, enhanced protection against DNA damage, and improved retinal arteriolar dilation. Furthermore, significantly modified gene expression alterations were related to highly relevant biological processes such as DNA repair, signal transduction, and transcription.

Among these results, no immunity‐related genes or pathways, nor the proportion of five types of leukocytes, were found to be altered after the intervention, which is not consistent with previous findings from both in vivo and in vitro studies [13, 14, 15, 16]. We hypothesize that the constraints of analyzing heterogeneous white blood cell (WBC) populations hampered the detection of a prominent expression signal in the MiBLEND intervention. In this research, therefore, we want to use CIBERSORTx to enhance gene expression analysis, providing new insights into cell‐type‐specific changes of the leukocytes following the intervention. These may help us understand the responses of various immune cells and their communication that is contributed by different F&Vs, and establish the correlation between the regulated pathways and the function of specific cell types, indicating more detailed and precise conclusions.

## 2 Methods

### 2.1 Data Source

For this study, we used RNA sequencing data of blood samples from 145 participants of the MiBLEND study [12]. This dietary intervention study includes healthy 40 males and 105 females with an average age of 27 + 10 years and a BMI of 22.7 ± 2.2 kg/m^2^. The MiBLEND study was powered to detect a 20% effect size at FDR < 0.05 with 80% power using the PowerAtlas tool [17], aiming for 40 participants per blend group. As our analysis used all available data from this trial, no new sample size calculation was performed. The dietary intervention included seven different blends, and their composition is listed in Table S1. The expected daily intake of key phytochemicals per blend (e.g., carotenoids, vitamin C [VitC], quercetin, and catechins) was quantified based on ingredient content and previously published values. These values are provided in Figure S1 of the original MiBLEND publication [12]. The participants were randomly assigned to consume two of seven different blends of F&V (at 450 g/day) for 2 weeks. Before the first intervention, there was a 2‐week washout with a low amount of F&V (50 g/day), while there is 1‐week of the same washout between interventions. For all participants, we collected data on WBCs composition, including the proportion of neutrophils, eosinophils, basophils, monocytes, and lymphocytes. Furthermore, we used the following outcome parameters: (1) oxidative stress markers including %Tail moment (TM) and %Tail DNA (TDNA) test by comet assay, Trolox‐equivalent antioxidant capacity (TEAC), ROS (ROSHeight and ROSArea) tested by electron spin resonance (ESR), 8‐hydroxyguanosine (8‐OHdG), and 8‐isoprostane (8‐iso‐PG); (2) retinal microvasculature parameters including central retinal artery equivalent (CRAE), central retinal vein equivalent (CRVE), arteriolar‐to‐venular ratio (AVR), vessel tortuosity (TORT), and vessel branching (BRAN); (3) circulating phytochemicals included total polyphenols (TPs), carotenoids (α‐carotene and β‐carotene), VitC, lutein, and lycopene.

Further details regarding the measurement of weight, height, BMI calculation, laboratory assays, and equipment used in the MiBLEND intervention have been described in full in DeBenedictis et al. [12]. In brief, height and weight were measured under fasting conditions using standardized equipment, and BMI was calculated as weight (kg)/height^2^ (m^2^) [18].

### 2.2 Data Preparation

Preprocessing of the RNA sequencing data from the BCL file was done according to the initial data analyses in the MiBLEND study [12]. No outlier samples were identified as being more than 20% different than another sample. Six samples were removed because their total read count was below the 3‐million‐read threshold. This resulted in 383 samples that were used for subsequent analyses. A total of 46 664 genes were removed due to having less than 1 CPM (counts per million), leaving 10 577 remaining genes.

### 2.3 CIBERSORTx

Processed and CPM [19] normalized mRNA sequencing data from blood samples of the participants in the MiBLEND study were uploaded to CIBERSORTx [10, 11] (http://cibersortx.stanford.edu) in order to generate fractions of 22 types of leukocytes (Figure S1), based on the LM22 Signature Matrix file from CIBERSORTx website. The group mode of CIBERSORTx was used to generate cell‐type‐specific gene profiles with B‐mode batch correction and LM10_merged_classes file, which merged the 22 types of cells to 10 types: B cells, plasma cells, CD8+ T cells, CD4+ T cells, natural killer (NK) cells, monocytes, dendritic cells (DCs), mast cells, eosinophils, and neutrophils.

### 2.4 Paired *t* Test and Correlation Analysis

A paired *t* test was performed between the proportion of WBCs of the participants before and after the intervention with different blends of F&Vs, and graphs were generated for comparisons with *p* values ≤ 0.05 in GraphPad Prism 8.0.2 (San Diego, CA, USA, www.graphpad.com). Data are presented as mean ± SEM unless otherwise stated. In order to validate the different cell fractions generated in CIBERSORTx, the proportion of lymphocytes and monocytes was firstly generated by summing up all their subtypes’ proportion in CIBERSORTx, which, along with the neutrophil fraction, was compared with that of the Maastricht University Medical Center+ (MUMC⁺) results by Pearson correlation coefficient. Pearson's correlation analysis was also performed to examine the associations between cell fractions with oxidative stress markers, retinal microvasculature parameters, and phytochemical levels in circulation. Before this correlation analysis, variables were assessed for normality using the Shapiro–Wilk test [20]. Only variables showing nonidentical values and satisfying the assumption of normal distribution (*p* > 0.05) were included in the correlation analysis to ensure the validity of parametric testing.

### 2.5 Selection of Differentially Expressed Genes and Pathway Enrichment

Log_2_FC was generated as previously described [21, 22], by “LogFC = log((geps2$EEC + 1)/(geps1$EEC + 1),2)”, where geps2 refers to gene expression value after the dietary intervention with F&V Blends 1, 2, 3, 5, 6, or 7, while geps1 refers to the baseline measurement. FDR‐adjusted *p* values using the Benjamini–Hochberg method [23] were calculated to correct for multiple comparisons in R version 4.2.1 (R Core Team, 2022) [24]. Then, DEGs were identified using the cutoff thresholds of |log2FC| > 1 and FDR < 0.05. Lastly, the DEGs were uploaded to Cytoscape software (version 3.9.1) [25] for pathway enrichment analysis using ClueGO [26] and CluePedia [27] apps with the latest KEGG [28] pathway database (cutoff threshold of BH adjusted *p* < 0.05). Grouped terms generated by ClueGO during KEGG pathway enrichment represent clusters of functionally related pathways that are significantly overrepresented. KEGG mapping tool [29] was used to show the subpathways regulated with labeling of the involved DEGs.

## 3 Results

### 3.1 Investigation of Changes in Cell‐Type Proportion and Validation of the Fractions Generated by CIBERSORTx

To explore the impact of different F&V interventions on cell‐type proportions in human blood, we quantified the proportions of the five types of leukocytes from clinical total WBC count data. Neutrophils showed the highest proportion, followed by lymphocytes and monocytes, with basophils being the least prevalent among the cell types. Comparisons across intervention groups (DI1–DI7, Dietary Intervention with Blends 1–7) and the baseline (DI0) revealed no significant differences in cell proportions (Figure S2A). Pearson's correlation was used to check the effect of sex, age, and BMI. The absolute *R* values for all were between 0 and 0.2, indicating no significant correlation. As the number of participants for DI4 is relatively low, DI4 was removed in the next analysis.

**FIGURE 1:**
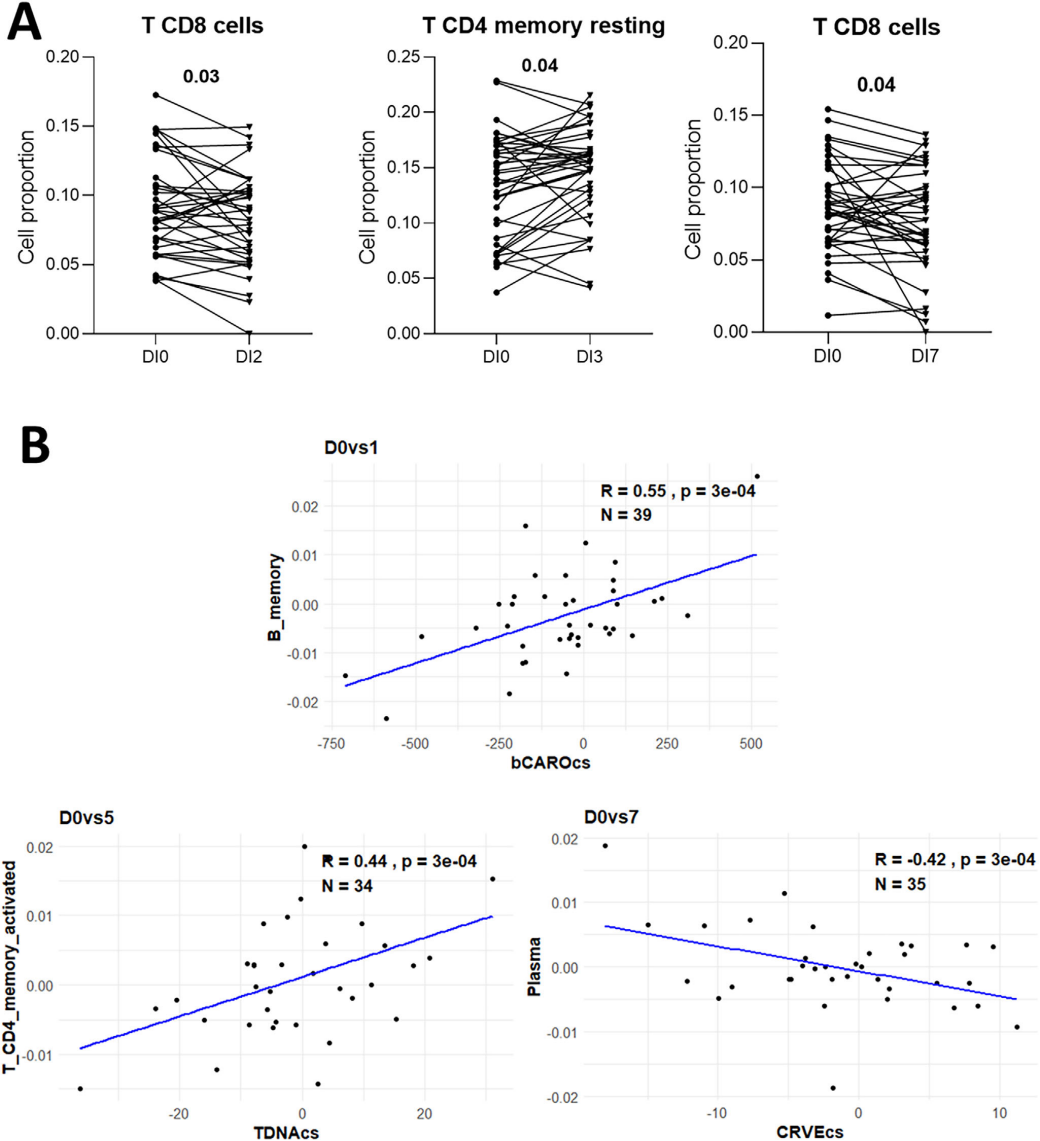
Significant changes in cell proportions postintervention and their correlation with alterations in oxidative stress, retinal microvasculature, and phytochemical levels in circulation. (A) Graph illustrating significant changes in the proportions of three cell types postintervention, identified using paired *t* tests (*p* values of 0.03 and 0.04). (B) Scatter plots illustrating the four highly and significantly correlated changes in cell proportions and markers (|*R*| > 0.4, *p* < 0.01).

For a detailed analysis of sub‐cell types, we utilized the CIBERSORTx pipeline to generate fractions for 22 types leukocytes, based on the LM22 signature matrix (Figure S2B). Consistent with the clinical data, neutrophils remained the most abundant. Besides, there were almost no activated mast cells or gamma delta T cells. We validated these results by analyzing the correlation between the fractions obtained from clinical data and CIBERSORTx, which showed a strong positive correlation (*R* = 0.886; *p* value = 2.5e‐322; Figure S2C). Notably, CD8 T cells significantly decreased following interventions with Blend 2 (Blend 0: 9.1% ± 0.57%, Blend 2: 8.2% ± 0.56%, *p* value = 0.03) and Blend 7 (Blend 0: 8.7% ± 0.51%, Blend 7: 7.7% ± 0.56%, *p* value = 0.04), while memory resting CD4 T cells increased postintervention with Blend 3 (Blend 0: 13.4% ± 0.75%, Blend 3: 14.56% ± 0.66%, *p* value = 0.04), as determined by paired *t* tests (Figure 1A).

### 3.2 Correlations Between Immune Cell Proportion Changes and Clinical Markers

For the biological implications of the immune cell fraction changes, Pearson's correlation analysis was employed to examine the associations between these changes and oxidative stress markers (TMcs, TEACcs, TDNAcs, ROSHeightCS, ROSAreaCS, OHDG8cs, and 8‐iso‐PGcs), retinal microvasculature parameters (CRAEcs, CRVEcs, AVRcs, TORTcs, and BRANcs), and circulating phytochemicals (TPcs, aCAROcs, bCAROcs, VitCcorrCS, LuteinCS, and LycopeneCS) (Figure S3) after normality assessment (Supporting Information File S1).

**FIGURE 2:**
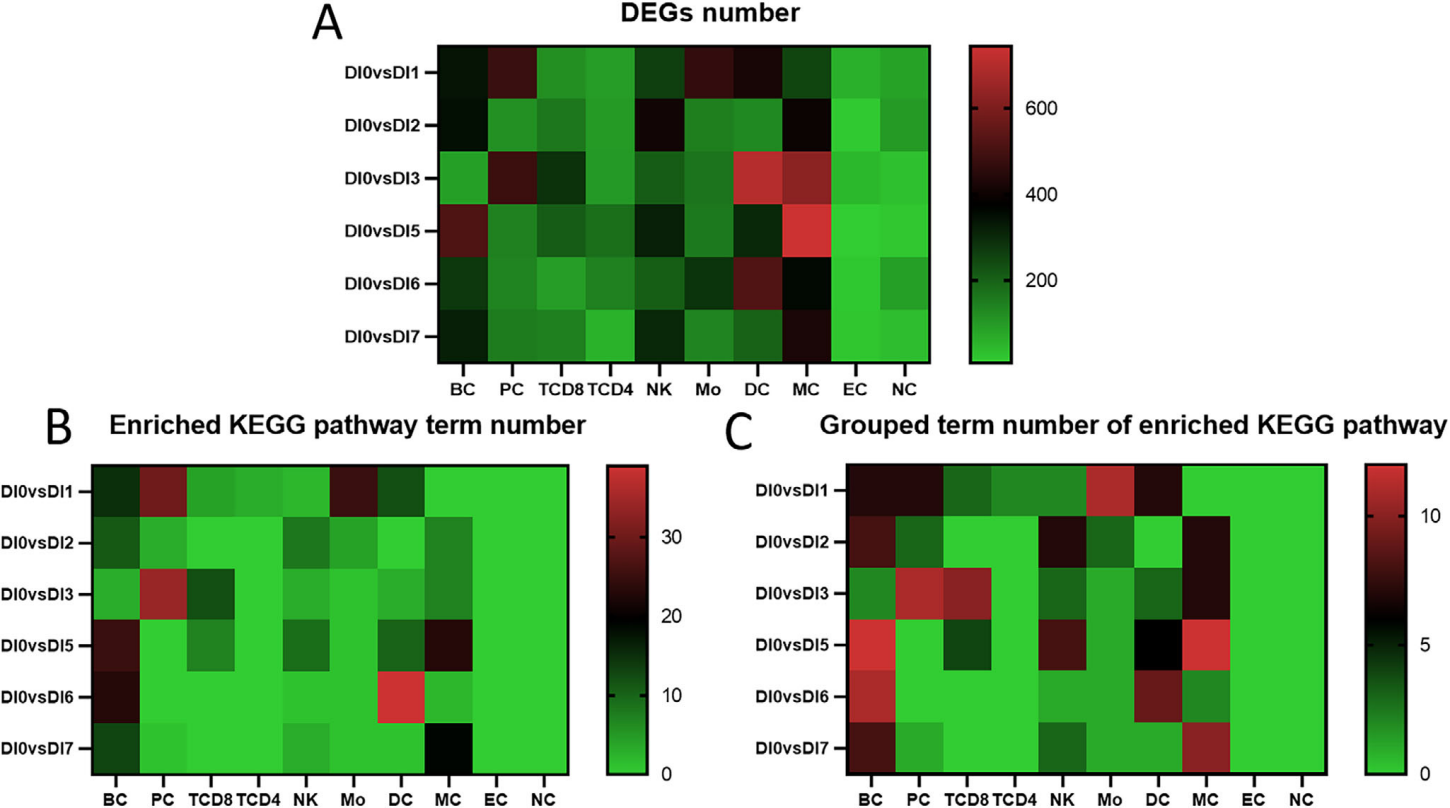
Summary of transcriptomic changes following fruit and vegetable (F&V) interventions. (A) Heatmap illustrating the number of differentially expressed genes (DEGs) postintervention for each of the six F&V blends compared to the baseline DI0. (B) Heatmap displaying the number of KEGG pathways enriched with DEGs for each blend, using a significance threshold of BH‐adjusted *p* ≤ 0.05. (C) Heatmap showing the number of grouped terms within the enriched KEGG pathways. Abbreviations of leukocyte subsets: B cells (BC), plasma cells (PC), CD8⁺ T cells (TCD8), CD4⁺ T cells (TCD4), natural killer (NK) cells, monocytes (Mo), dendritic cells (DC), mast cells (MC), eosinophils (EC), and neutrophils (NC).

Our results revealed three significant correlations (|*R*|> 0.4, *p* < 0.01) as depicted in Figure 2A. Changes in memory B cell proportions were strongly positively correlated with changes in plasma β‐carotene levels following the Blend 1 intervention (*R* = 0.55, *p* value = 3e‐04). Changes in CD4+ activated memory T cells were positively correlated with TDNA changes post‐Blend 5 intervention (*R* = 0.44, *p* value = 3e‐04). Conversely, plasma cell (effector B cell) changes were negatively correlated with CRVE changes after the Blend 7 intervention (*R* = 0.42, *p* value = 3e‐04), as shown in Figure 1B.

### 3.3 Cell‐Type‐Specific Transcriptomic Changes Following F&V Interventions

The group mode of CIBERSORTx was utilized to generate cell‐specific gene profiles. Due to the limited sample size for each blend, the 22 cell types were consolidated into 10 major types: B cells, plasma cells, CD8+ T cells, CD4+ T cells, NK cells, monocytes, DCs, mast cells, eosinophils, and neutrophils. Differentially expressed genes (DEGs) were identified using a threshold of FDR < 0.05 and an absolute log_2_ fold change greater than 1. Figure 2A, a heatmap, illustrates the distribution of DEGs for each cell type following every intervention, with values ranging from 0 to 800. Eosinophils and neutrophils exhibited the fewest DEGs, whereas mast cells, B cells, and DCs had relatively higher numbers of DEGs.

Subsequently, the number of DEGs across enriched KEGG pathways was summarized in Figure 2B (ranging from 0 to 40), and the total number of grouped terms within the enriched KEGG pathways as shown in Figure 2C (ranging from 0 to 15). Neither eosinophils nor neutrophils showed enriched KEGG pathways. B cells displayed a high number of enriched pathways across all interventions, except for Blend 3. Plasma cells exhibited a higher number of enriched pathways predominantly after interventions with Blends 1 and 3. Monocytes showed enrichment after Blend 1, DCs after Blend 6, CD8+ T cells after Blend 3, and mast cells after Blends 5 and 7. Overall, these results indicate distinct transcriptomic responses among different cell types to various blend interventions.

**FIGURE 3:**
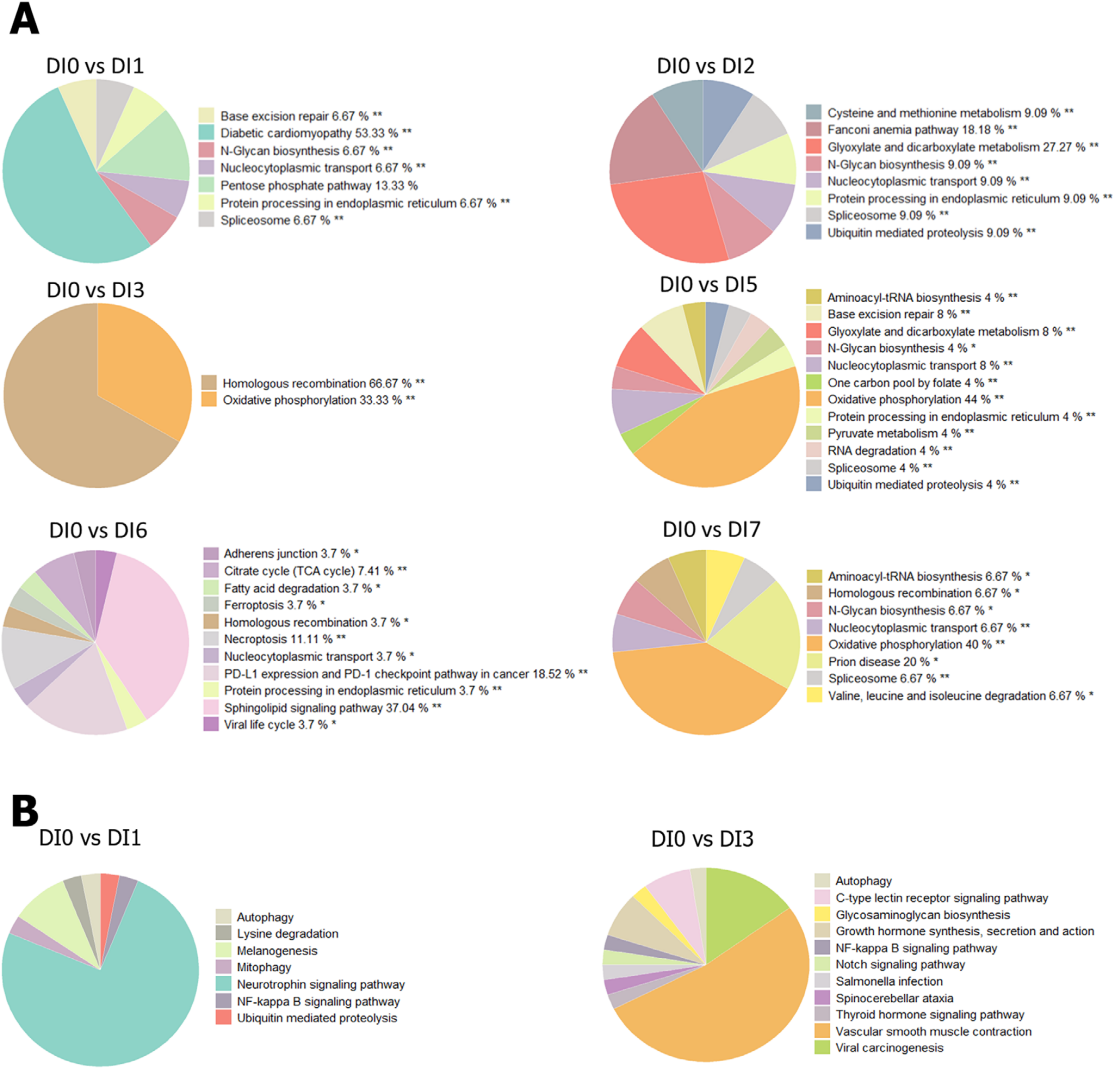
Enriched KEGG pathways in B cells and plasma cells following different fruit and vegetable (F&V) blend interventions. (A) Pie charts summarizing grouped KEGG pathway terms significantly enriched in B cells, defined here as a combined cluster of native and memory B cells, after each F&V blend intervention. (B) Pie charts showing enriched pathway groups in plasma cells, representing effector B cells, following intervention with Blends 1 and 3. Percentages represent the proportion of pathways within each group relative to the total number of enriched pathways. * Adjusted group *p* < 0.05; ** adjusted group *p* < 0.01.

**FIGURE 4:**
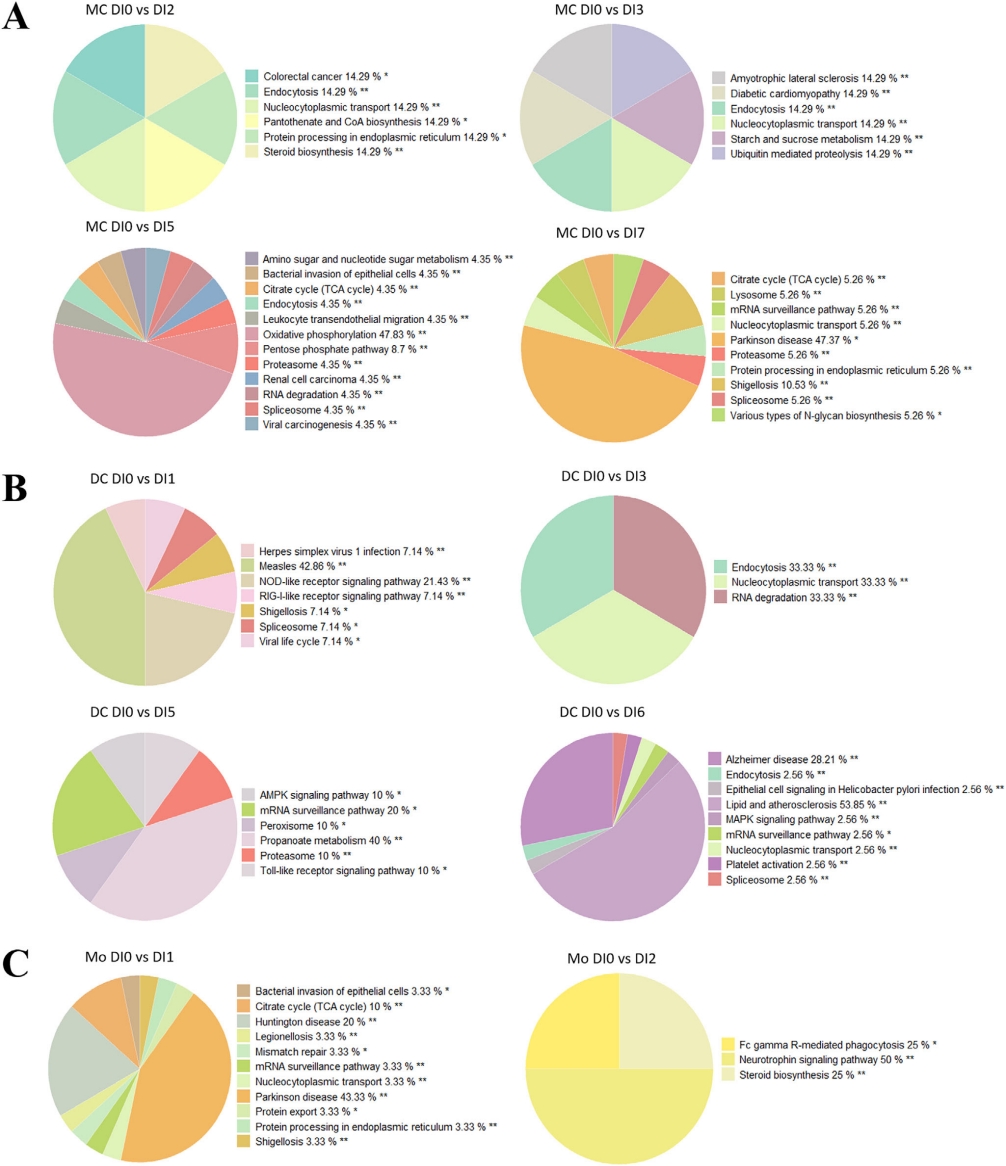
Altered pathways in antigen‐presenting cells (APCs) after different fruit and vegetable (F&V) blend interventions. (A) Pie charts show the grouped terms of the enriched KEGG pathways mast cells after intervention with Blends 2, 3, 5, and 7; (B) that in dendritic cells after intervention with Blends 1, 3, 5, and 6; (C) and that in monocyte after intervention with Blends 1 and 2 (** adjusted group *p* < 0.01, * adjusted group *p* < 0.05).

**FIGURE 5:**
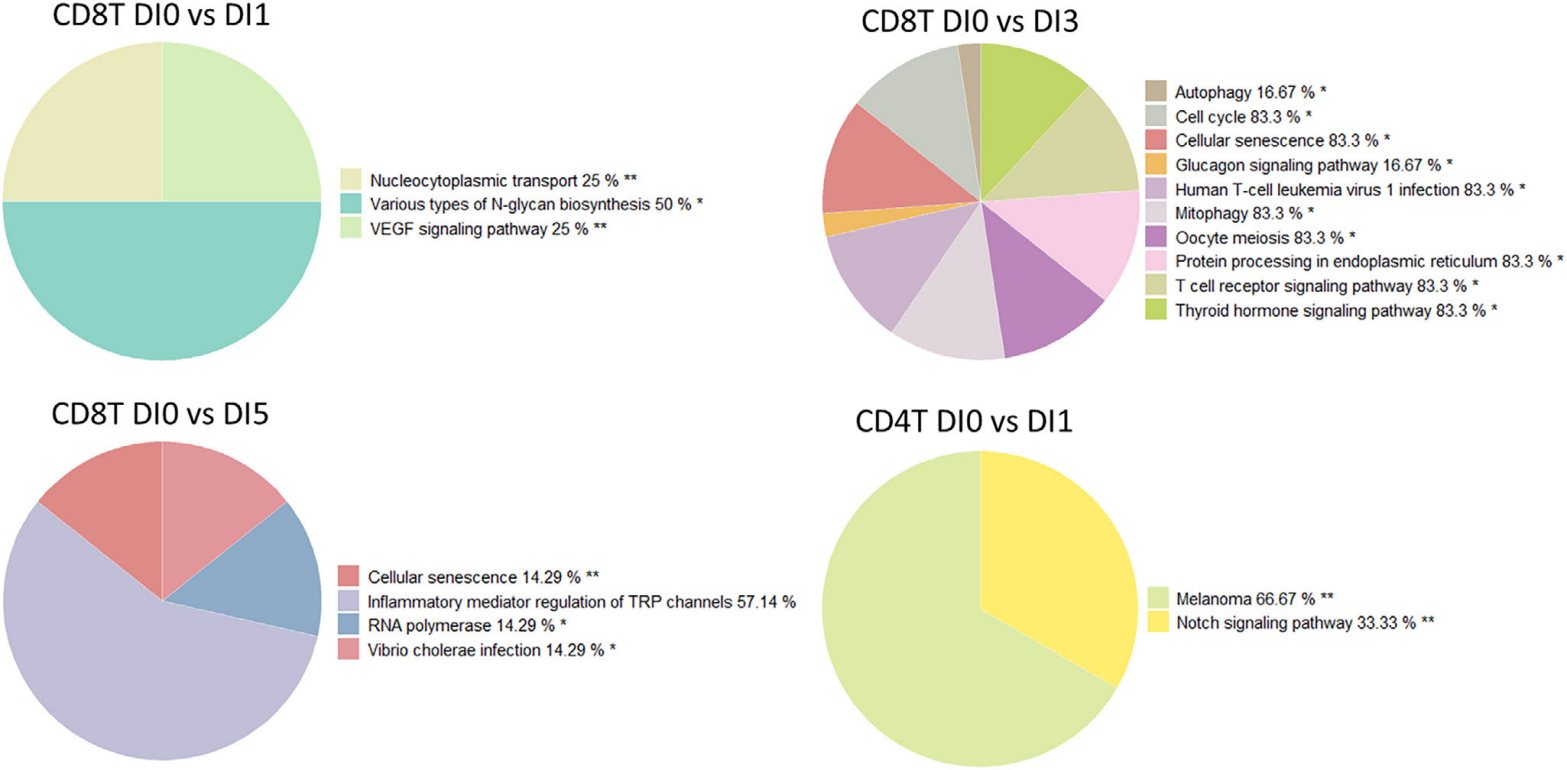
Altered pathways in T cells after different fruit and vegetable (F&V) blend interventions. Pie charts show the grouped terms of the enriched KEGG pathways in T cell after intervention of Blends 1, 3, and 5 (** adjusted group *p* < 0.01, * adjusted group *p* < 0.05).

**FIGURE 6:**
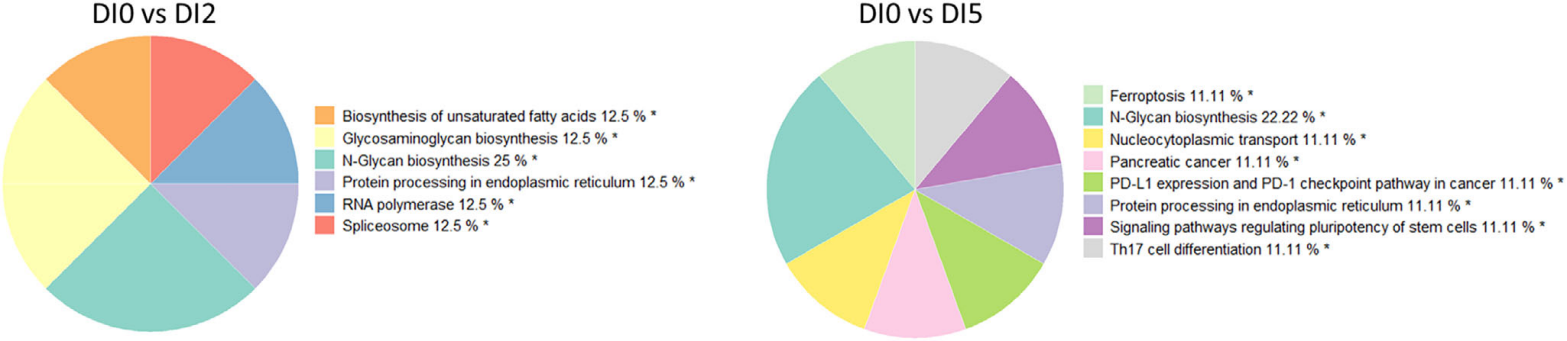
Altered pathways in natural killer (NK) cells after different fruit and vegetable (F&V) blend interventions. Pie charts show the grouped terms of the enriched KEGG pathways in (A) NK cell after intervention of Blends 2 and 5 (** adjusted group *p* < 0.01, * adjusted group *p* < 0.05).

Furthermore, pie charts in Figures 3, 4, 5, 6 were used to show the group terms of enriched pathways in each cell type after intervention using GlueGO in Cytoscape, highlighting the most frequently occurring and specific pathways for each cell type. The KEGG map in Figures S4–S5 was used to show the involved gene changes after different blends.

#### 3.3.1 B Cell (Native and Memory B Cell): DNA Repair

For B cells, DNA repair pathways such as base excision repair (BER) and homologous recombination (HR) were enriched by DEGs after intervention with almost every blend, except for Blend 2, while the nucleocytoplasmic transport pathway was also enriched after almost every blend except for Blend 3. The N‐glycan biosynthesis pathway was enriched after intervention with Blends 1, 2, 5, and 7 (Figure 3A). The DEGs involved in BER after Blend 1 included PARP, TDG, and APEX, while the ones after Blend 5 were PARP and Polδ/ε. These genes were all upregulated and engaged in the two main types of repair on the oxidized/ring‐saturated base and deaminated, alkylated, or mismatched base (Figure S4A). The DEGs involved in HR included Rad51C, BLM, and Mus81 after Blend 3, Mus81, and polδ after Blend 6, Mus81, polδ, and DSS1 after Blend 7. These genes were also upregulated, and engaged in both double‐strand break repair (DSBR) and synthesis‐dependent strand annealing (SDSA) (Figure S4B).

#### 3.3.2 Plasma (Effector B Cells): NF‐kappaB Signaling

Plasma cells had quite a lot of DEGs and enriched pathways only after the intervention of Blends 1 and 3. The pie chart of the grouped terms showed that the NF‐kappaB signaling pathway was enriched after both Blends 1 and 3. The number of pathways in the neurotrophin signaling pathway group was highest (75%) after Blend 1, while those in the vascular smooth muscle contraction group were highest (52.5%) after Blend 3 (Figure 3B). Within the NF‐kappaB signaling pathway, genes such as ATM, ELKS, e‐FLIP, TRIM25, TIRAP, and CK2 were all regulated after Blend 1, while genes XIAP, CYLD, p50, LIGHT, LT‐βR, NIK, TIRAP, and CK2 were regulated after Blend 3. The relatively fully regulated subpathway LIGHT‐LT‐βR‐NIK, which is the upstream part of the noncanonical pathway, was related to B cell development and survival (Figure S4C).

#### 3.3.3 Antigen‐Presenting Cells (APCs)

In the immune system, APCs play a crucial role in initiating and modulating immune responses. APCs are responsible for capturing, processing, and presenting antigens to T cells, thereby bridging innate and adaptive immunity. Among the primary APCs are mast cells, DCs, and monocytes. Each of these cell types has unique characteristics and functions, and the following results highlight their specific altered pathways after intervention with certain blends.

##### 3.3.3.1 Mast Cells: Endocytosis

Mast cells were mainly regulated at gene level after intervention with Blends 2, 3, 5, and 7, and the endocytosis pathway was enriched after Blends 2, 3, and 5. Disease‐related pathways were more frequently enriched in mast cells such as the colorectal cancer pathway after Blend 2, the diabetic cardiomyopathy pathway after Blend 3, and the Parkinson disease pathway after Blend 7 (47.37%) (Figure 4A). The DEGs involved in the endocytosis pathway after the intervention were almost exclusively involved in clathrin‐dependent endocytosis (Figure S5A).

##### 3.3.3.2 Dendritic Cells: Receptor Signaling Pathway and Endocytosis

DCs’ pathways were mainly regulated after the intervention with Blends 1, 3, 5, and 6. Among these, the receptor signaling pathways, such as RIG‐I‐like receptor (RLR) signaling pathway and NOD‐like receptor (NLR) signaling pathway, were enriched after intervention with Blend 1, while the Toll‐like receptor (TLR) pathway was enriched after intervention with Blend 5. Meanwhile, the endocytosis pathway was enriched both after intervention with Blends 3 and 6 (Figure 4B). The difference between DCs and mast cells is that clathrin‐independent endocytosis was regulated in DCs with the involvement of DEGs such as IL‐2R and Src (Figure S5B). Figure S5C also shows that the CD86 gene directly contributes to T cell stimulation, which was regulated after intervention with Blend 5. Furthermore, DEGs after the intervention with Blend 1, such as DAK, RIG‐I, MDA5, MITA, NAP1, and MEKK1, were involved in the upstream pathway of the RLR signaling pathway, which may contribute to the activation of DCs and NK cells, as well as antibody production (Figure S5D).

##### 3.3.3.3 Monocytes: Fc Gamma R‐Mediated Phagocytosis

In monocyte cell, disease‐related pathways in the group term Parkinson disease were enriched after intervention with Blend 1, while neurotrophin signaling pathway and Fc gamma R‐mediated phagocytosis pathways were enriched after intervention with Blend 2 (Figure 4C).

#### 3.3.4 T Cell: TCR Signaling Pathways

Directly related immune pathways in CD8+ T cells such as T cell receptor (TCR) signaling pathway and human T cell leukemia virus 1 infection were enriched after intervention with Blend 3, and the inflammatory mediator regulation of TRP channels pathway was enriched after intervention with Blend 5 (Figure 5). After the intervention with Blend 1, pathways in various types of the N‐glycan biosynthesis group were mainly enriched in CD8+ T cells. In CD4+ T cells, only the Melanoma and the Notch signaling pathway were enriched (Figure 5). Among the DEGs after Blend 3, genes involved in immune pathways such as AKT2, ATF2, ATF4, CHEK2, IL15RA, NFATC3, NFYB, and GRAP2 were upregulated, while the genes ANAPC4, MAD1L1, and CD8B were downregulated (Figure S6).

#### 3.3.5 Natural Killer Cell

N‐glycans biosynthesis group term was enriched in NK cell both after intervention with Blends 2 and 5. Immune‐related pathway like PD‐L1 expression and PD‐1 checkpoint pathway in cancer pathway and Th17 cell differentiation were also enriched in NK cell after intervention with Blend 5 (Figure 6).

## 4 Discussion

In this study, we investigated cell‐type‐specific proportions and gene expression changes in human blood following interventions with various F&V blends, examining their correlations with oxidative stress‐induced DNA damage and retinal microvasculature alterations. We found no significant changes postintervention on the proportion of basophils, eosinophils, lymphocytes, monocytes, and neutrophils by analyzing results from clinical WBC counts. In order to obtain insights into cell‐specific transcriptomic changes based on mRNA sequencing data from the MiBLEND study, we applied the CIBERSORTx deconvolution method. The results of this analysis were found to be well validated by cell fractions measured by the clinical diagnostics lab using standard hospital practices. Remarkably, the proportion of CD4+ memory resting T cells increased in participants who consumed Blend 3, which contains the highest levels of alpha‐carotene, beta‐carotene, and VitC among the blends tested [12]. Also, previous research has shown that high intake of beta‐carotene can increase CD4+ T cell counts in humans (60 mg/day) [30] and total T cell counts in the elderly (45 mg/day) [31], but did not investigate changes within the finer subcategories of CD4+ T cells. CD4+ memory resting T cells, which develop from effector T cells after primary immune responses and survive pathogen clearance, provide a rapid and robust response upon re‐encounter with the same or similar antigens [32]. Thus, the observed increase in the proportion of these cells suggests that Blend 3 may enhance the immune system's ability to respond more quickly to recurrent health challenges.

Conversely, the proportion of CD8+ T cells significantly decreased following interventions with Blends 2 and 7. Blend 2 contains the highest concentrations of anthocyanins, specifically delphinidin‐3‐glucoside, cyanidin‐3‐glucoside, and peonidin‐3‐glucoside, while Blend 7 features the most diverse combination of ingredients. Anthocyanins have been shown to boost CD8+ T cell infiltration in tumors, with or without other treatments [33, 34].

Interestingly, although Blend 1 does not contain carotenoids, only in this intervention did changes in plasma β‐carotene levels strongly correlate with changes in memory B cell proportions (*r* > 0.5). This association was not observed following other interventions, including those with carotenoid‐rich blends (e.g., Blend 3 or Blend 7), suggesting that the immunological impact of β‐carotene may be context‐dependent. A plausible explanation is that the flavonoid‐rich composition of Blend 1, particularly quercetin and catechins, may modulate the intestinal redox environment and potentially enhance the absorption and metabolic conversion of β‐carotene to retinoic acid, a bioactive metabolite known to influence B cell differentiation and memory formation. Although we did not directly measure retinoic acid levels in plasma, previous studies have shown that quercetin can inhibit the activity of β‐carotene‐15,15′‐dioxygenase, the key enzyme responsible for converting β‐carotene into retinal, thereby affecting its metabolic fate in intestinal cells [35, 36]. Flavonoids also exert antioxidant effects within the intestinal mucosa, which may further support a favorable redox environment for nutrient absorption [37]. Retinoic acid has been shown to support B cell survival and class switching and to facilitate memory B cell development through T follicular helper (Tfh) cell interactions and epigenetic modulation [38, 39]. Moreover, flavonoids themselves may provide an antiinflammatory and oxidative balance favorable for sustaining memory B cells [40]. Quercetin and fisetin have also been reported to enhance the intestinal transport and plasma bioavailability of catechins, supporting a model of cooperative absorption among phytochemicals [41]. The lack of such correlation in other blends, despite higher carotenoid content, may be due to competing phytochemicals (e.g., isothiocyanates and allicin) or more complex food matrices interfering with carotenoid bioavailability or signaling. In particular, isothiocyanates, such as allyl isothiocyanate (AITC), have been shown to enhance the Z‐isomerization and decomposition of carotenoids, including lycopene and β‐carotene, during heat treatment [42]. This effect may lead to a change in the bioavailability of carotenoids, as Z‐isomers are typically more bioavailable than their all‐trans counterparts. Moreover, while polysulfides can improve the thermal stability of carotenoids, thus preventing their breakdown, the combined presence of multiple phytochemicals in complex food matrices may alter carotenoid stability and absorption efficiency. These findings suggest that competing phytochemicals, particularly isothiocyanates, may indeed interfere with carotenoid bioavailability by promoting Z‐isomerization and possibly altering their stability and metabolic pathways.

Subsequent analysis of DEGs and enriched pathways indicated specific responses in each cell type to the intake of various blends, closely linked to their inherent characteristics and functions. Particularly, B cells (both native and memory) are the most responsive, as nearly all blends trigger numerous regulated pathways in these cells, with the exception of Blend 3. Predominantly, DNA repair pathways such as BER [43] and HR [44] were highlighted, which were both reported crucial not only for mitigating DNA damage and mutations but also for their roles in B‐cell mutagenesis during processes like immunoglobulin class switch recombination and somatic hypermutation. Additionally, immunoglobulin D switching has been reported to occur through HR in human Β cells [45]. Actually, the DNA repair pathway was identified as one of the main enriched pathways after the intervention in bulk blood RNA sequencing data analysis. Therefore, the upregulation of the engaged gene PARP, APEX, polδ, DSS1, BLM and Mus81 and TDG in B cells indicate that the intervention of various blends, except for Blend 2, may enhance the correction of error‐prone processes at the Ig loci in B cell (both native and memory) to reduce the risk of B‐cell lymphomas, and to support the Ig class switch. Meanwhile, plasma cells (effector B cell) were highly affected by Blends 1 and 3 at the gene level with the NF‐kappa B signaling pathway regulated after both blends’ intervention. This pathway's activation mediates B cell class‐switching to generate IgG, IgA, and IgE, and supports the differentiation of B cells into plasma cells that secrete antibodies at a high rate, as well as survival of plasma cells [46]. Finally, survival‐related genes cFLIP [47] and XIAP [48] were upregulated in plasma cells after intervention with Blends 1 and 3, respectively. The LIGHT‐LT‐βR‐NIK axis was also upregulated after intervention with Blend 3, which is crucial for mature B cell survival [49, 50]. Thus, consumption of Blends 1 and 3 may contribute to maintaining a robust immune system by improving plasma cell survival through NF‐kappa B signaling.

Mast cells were the second most responsive cell type following B cells, with numerous regulated pathways. Blends 2, 3, and 5 all regulated the endocytosis pathway in mast cells, which is pivotal for controlling allergic responses and preventing excessive inflammatory damage [51], as well as the process to present antigens on their surface using MHC Class II molecules to activate T cell [52]. Similarly, DCs also have their endocytosis pathway influenced by Blends 3 and 6, essential for T cell activation through the display of processed antigens on MHC molecules [53]. Unlike in mast cells, DCs’ endocytosis here includes clathrin‐independent routes facilitated by DEGs such as IL‐2R and Src that are associated with TLR signaling [54]. Additionally, various receptor pathways, including RLR signaling pathways and NLR signaling pathway, were regulated in DC after intervention with Blend 1, while TLR pathway regulation was enriched after Blend 5. The RLRs in DC are responsible for prompting antiviral defense and maturation of DC [55, 56]. Activation of NLRs leads to maturation of DC and production of cytokines [57]. The TLRs contribute to the activation and maturation of DC [58], and the Blend 5‐regulated CD86 gene here can directly contribute to T cell stimulation by bounding CD28 [59]. Another crucial APC, the monocyte, exhibited a regulated phagocytosis pathway following the intervention with Blend 2. This pathway is of significant importance for the pathogen clearance and presentation function of the monocyte [60]. In summary, Blends 2, 3, and 5 can regulate antigen‐presenting function by affecting the clathrin‐dependent endocytosis in mast cell, while Blends 3 and 6 can affect both clathrin‐dependent and independent endocytosis of DC, along with receptor pathways influenced by Blends 1 and 5, thus contributing to the effective bridging of innate and adaptive immune responses. Blend 2 can regulate the pathogen clearance and presentation function of monocyte.

Correspondingly, gene expression changes in T cells demonstrated altered T cell signaling pathways after intervention with Blends 1, 3, and 5. TCR signaling pathway and human T‐cell leukemia virus 1 infection were enriched after intervention of Blend 3 in CD8+ T cell, while inflammatory mediator regulation of TRP channels was regulated after intervention with Blend 5. CD4+ T cell's Notch signaling pathway was regulated after intervention with Blend 1, in which Notch can colocalize with CD4 in TCR activation [61]. Additionally, various types of N‐glycan biosynthesis pathway were regulated by Blend 1 in CD8+ T cell, having a broad effect on the multiple T cell functions with impact both in autoreactivity and in immune tolerance [62]. In NK cells, Blends 2 and 5 also mainly affected N‐glycan biosynthesis, which can affect the antibody binding affinity and effector function of human NK cells [63]. Other cells like neutrophils and eosinophils did not show significantly regulated pathways after intervention of any blend.

Overall, our study demonstrates that consumption of specific F&V blends can modulate immune cell proportion and gene expression in human blood, as well as the pathways that play crucial roles in the immune function of each cell type. These findings highlight the potential of targeted dietary interventions to enhance cell‐specific immune functions and mitigate disease risk. Notably, Blend 3, rich in alpha‐carotene, beta‐carotene, and VitC, significantly increased the proportion of CD4+ memory resting T cells, suggesting enhanced immune readiness. Conversely, Blends 2 and 7, characterized by high anthocyanin content, reduced CD8+ T cell proportions, thereby potentially improving the inflammatory environment. Furthermore, correlations between changes in immune cell populations and retinal microvasculature parameters underscore the systemic impact of dietary components on vascular and ocular health. The pronounced gene expression changes observed, particularly in B cells and APCs, reveal the activation of DNA repair and endocytosis pathways, underscoring the nuanced cellular responses to these nutritional interventions. Besides, the application of CIBERSORTx deconvolution method in our study provided robust validation of cell‐type‐specific transcriptomic changes, as corroborated by clinical diagnostic lab measurements. This methodological approach has proven reliable and useful in deciphering complex cell population dynamics and gene expression profiles, enabling a deeper understanding of how nutritional interventions impact immune cell functions at a granular level. Collectively, our findings advocate for the strategic use of F&V blends to bolster immune defense mechanisms and reduce oxidative stress.

Although our results provide novel insights into immune cell‐specific responses to F&V blends, the absence of significant transcriptomic changes in certain cell types, such as neutrophils and eosinophils, raises questions about their threshold of responsiveness or the need for longer interventions. Furthermore, the observed modulation of antigen presentation and DNA repair pathways underscores the potential of dietary strategies not only in immune priming but also in reducing genomic instability, a hallmark of aging and cancer. Future studies should explore the long‐term effects of such interventions and assess their impact in populations with impaired immunity or chronic inflammation.

## 5 Limitation

Despite the strengths of this study, including the use of cell‐type‐specific transcriptomics and clinically validated immune cell profiling, several limitations should be acknowledged. First, the relatively short duration and moderate sample size of the intervention may limit the generalizability of our findings. Although the original MiBLEND [12] dietary intervention study employed a randomized crossover design with formal sample size calculation using the PowerAtlas [17] tool (targeting 80% power to detect 20% effect sizes at FDR < 0.05 with 40 participants per blend), the present analysis relied on available data from 145 participants and was not designed de novo. As a result, statistical power for specific subgroup analyses (e.g., DI4) may have been insufficient, especially given the unequal group sizes due to recruitment challenges during the COVID‐19 pandemic. Second, while CIBERSORTx enabled inference of cell‐type‐specific gene expression changes, its reliance on reference signatures and computational deconvolution may introduce bias [64]. Third, the associations observed between immune cell shifts, gene expression, and physiological endpoints are correlational in nature and do not establish causality. Additionally, while our study focused on pathway analysis to provide a broader understanding of immune responses, future studies using more precise methods such as single‐cell RNA sequencing or RT‐qPCR could provide more accurate measurements of specific gene expression changes, particularly in key genes that drive immune modulation. These techniques could offer more reliable validation of gene‐level changes across subsets and interventions. Fourth, although the phytochemical composition of each blend was quantified and published in Figure S1 of the original MiBLEND publication [12], the current study did not perform a stratified analysis of intervention order effects. Although the crossover design with washout periods was intended to minimize such effects, residual order‐related variation cannot be entirely excluded and warrants investigation in future studies. Finally, functional validation of the identified pathways in vitro or in vivo would further substantiate the mechanistic insights proposed in this study.

Additionally, the study was conducted during the COVID‐19 pandemic, which may have introduced behavioral, lifestyle, or immunological variability unrelated to the intervention. Although participants with recent infections or vaccinations were excluded, we cannot fully exclude the broader societal effects of the pandemic, such as changes in physical activity, stress, or dietary habits. Furthermore, our study cohort consisted primarily of young and healthy adults (mean age: 27 ± 10 years), which may limit the applicability of our findings to older populations or those with existing cardiovascular or immune‐related conditions. Future studies should explore these effects in more diverse populations, including elderly or clinically vulnerable groups.

## 6 Conclusion

In summary, this study demonstrates that consumption of specific F&V blends can modulate immune cell proportions and gene expression profiles in human peripheral blood in a cell‐type‐specific manner. Using deconvoluted transcriptomic data validated by clinical immune cell profiling, we identified key immunological pathways, including DNA repair and antigen presentation, as being responsive to different nutritional components. Notably, blends rich in carotenoids and VitC enhanced CD4⁺ memory resting T cell proportions, while anthocyanin‐rich blends altered CD8⁺ T cell populations, highlighting the functional specificity of these interventions. Furthermore, correlations between immune cell dynamics, retinal microvasculature changes, and oxidative stress markers emphasize the systemic impact of dietary modulation. These findings support the strategic use of nutritional interventions to enhance immune readiness and vascular health, and pave the way for future personalized nutrition strategies aimed at disease prevention and immune support.

## Ethics Statement

The study was approved by the local Medical Ethics Review Committee of the Maastricht University Medical Centre+ (MUMC+) (registration number: NL66118.068.18), registered at the International Trial Registry Platform (ICTRP) under identifier: NL7358, and conducted in accordance with the Declaration of Helsinki.

## Consent

All participants provided written informed consent before participation.

## Conflicts of Interest

The authors declare no conflicts of interest.

## Supporting information




**Supporting Information File S1**: mnfr70238‐sup‐0001‐SuppMat.xlsx

**Supporting Information File S2**: mnfr70238‐sup‐0002‐SuppMat.docx

## Data Availability

The RNA sequencing data will be made accessible in August 2025 under accession number E‐MTAB‐14563 (https://www.ebi.ac.uk/biostudies/arrayexpress/studies/E‐MTAB‐14563?key=32d50c10‐9d33‐4c71‐afc9‐2f8fdf84ab9b).
