# Supplementary material for: Unveiling Cell‐Type‐Specific Immune Reactions in Human Blood Following Varied Fruit and Vegetable Blends Interventions
Source: Mol Nutr Food Res. 2025 Sep 1;69(22):e70238. doi: 10.1002/mnfr.70238 (PMC12643190; doi:10.1002/mnfr.70238)
Supplement: Supplementary file 2 — Supporting Information File S2: mnfr70238‐sup‐0002‐SuppMat.docx [file MNFR-69-e70238-s002.docx]

# **Unveiling Cell-Type-Specific Immune Reactions in Human Blood Following Varied Fruit and Vegetable Blends Interventions**

Yueqin He^¶^, Julia N. DeBenedictis, Simone G. van Breda and Theo M. de Kok

Department of Translational Genomics, GROW – Research Institute for Oncology and Reproduction, Maastricht University, Maastricht, The Netherlands

**File list**

1. Supplementary Table 1
2. Supplementary Figure S1
3. Supplementary Figure S2
4. Supplementary Figure S3
5. Supplementary Figure S4
6. Supplementary Figure S5
7. Supplementary Figure S6


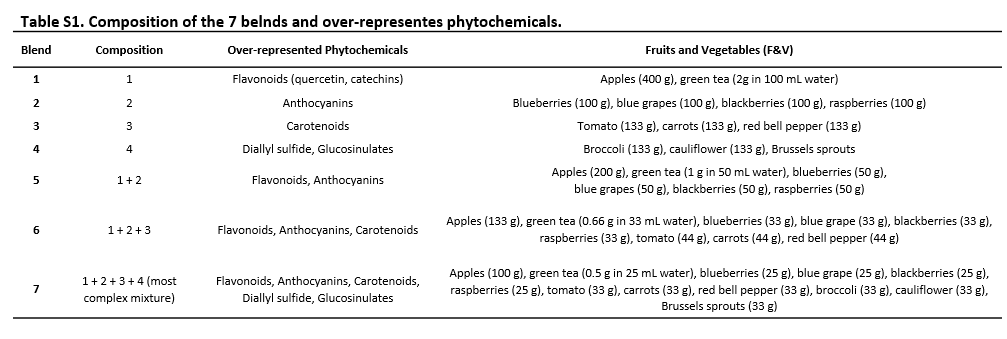


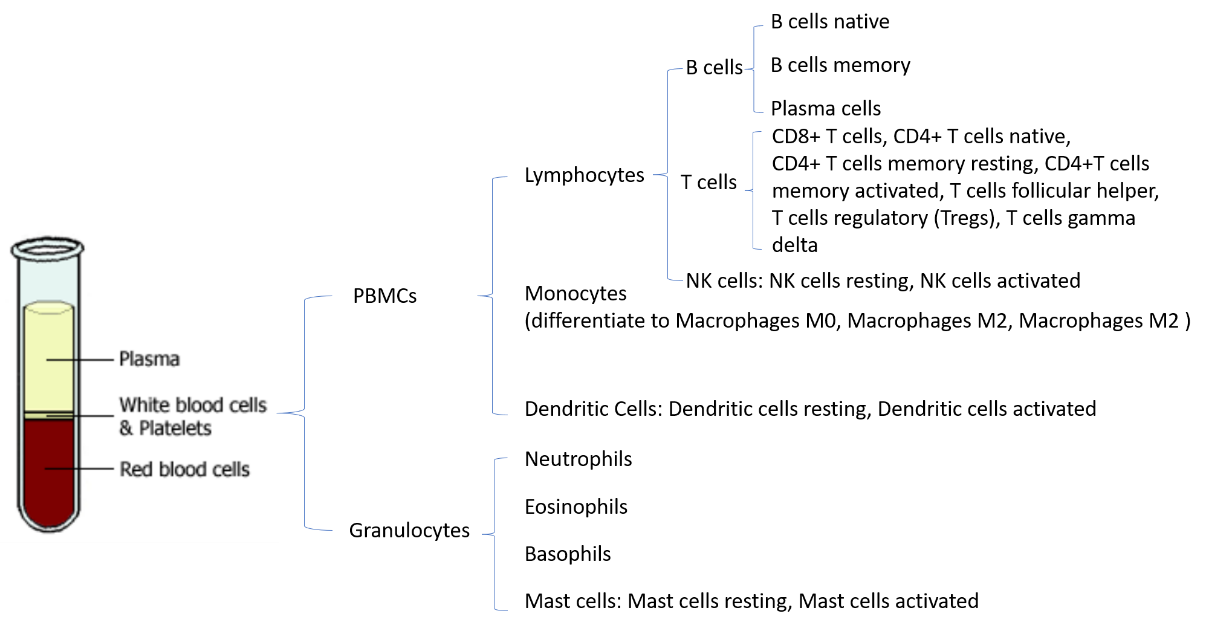


**Figure S1. The classification of major blood cell types and their respective subtypes.**


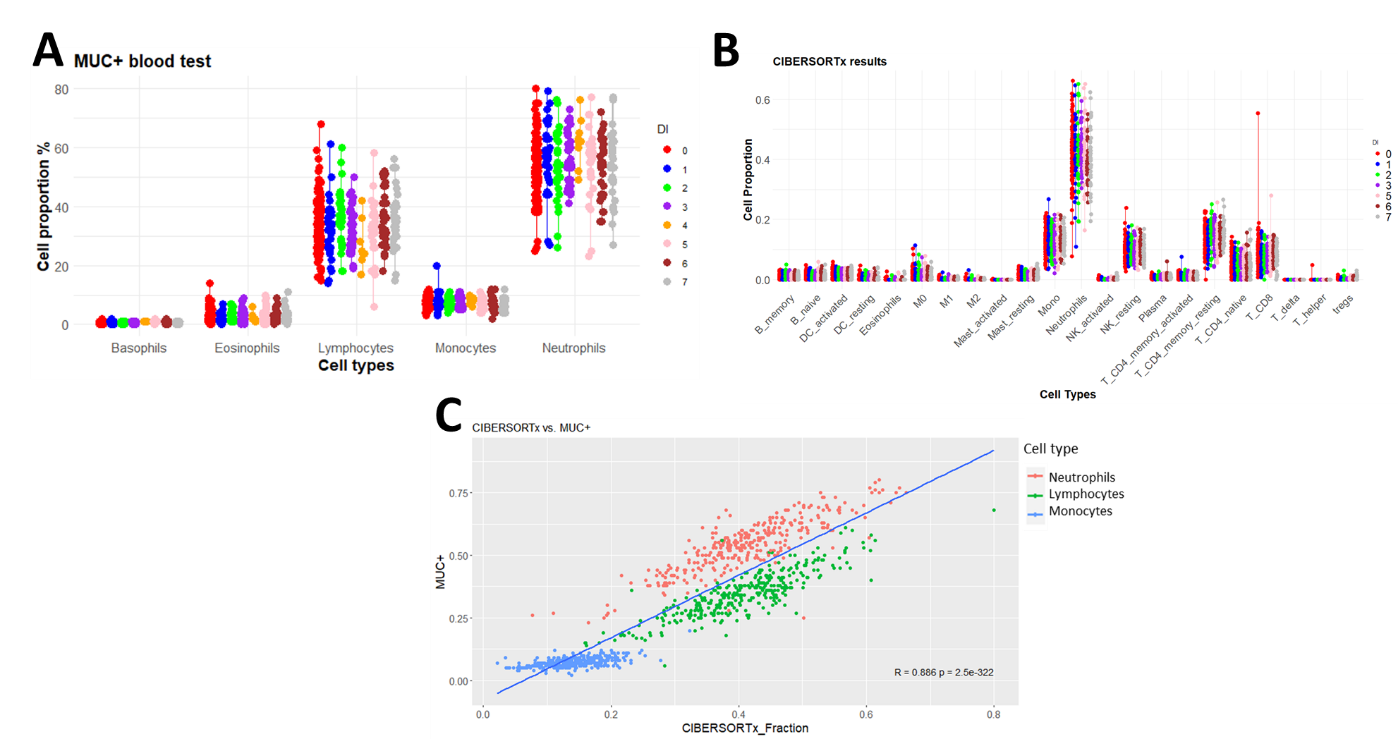


**Figure S2.** **Validation of blood cell type fractions generated by CIBERSORTx using clinical WBC data.** A) Dot plot showing the proportion of the five types of leukocytes in participants' blood as tested by clinical WBC. B) Dot plot of the distribution of 22 cell types in participants' blood as determined by CIBERSORTx. C) Scatter plot with trend line illustrating the correlation between cell proportions as determined by clinical data and CIBERSORTx analyses.


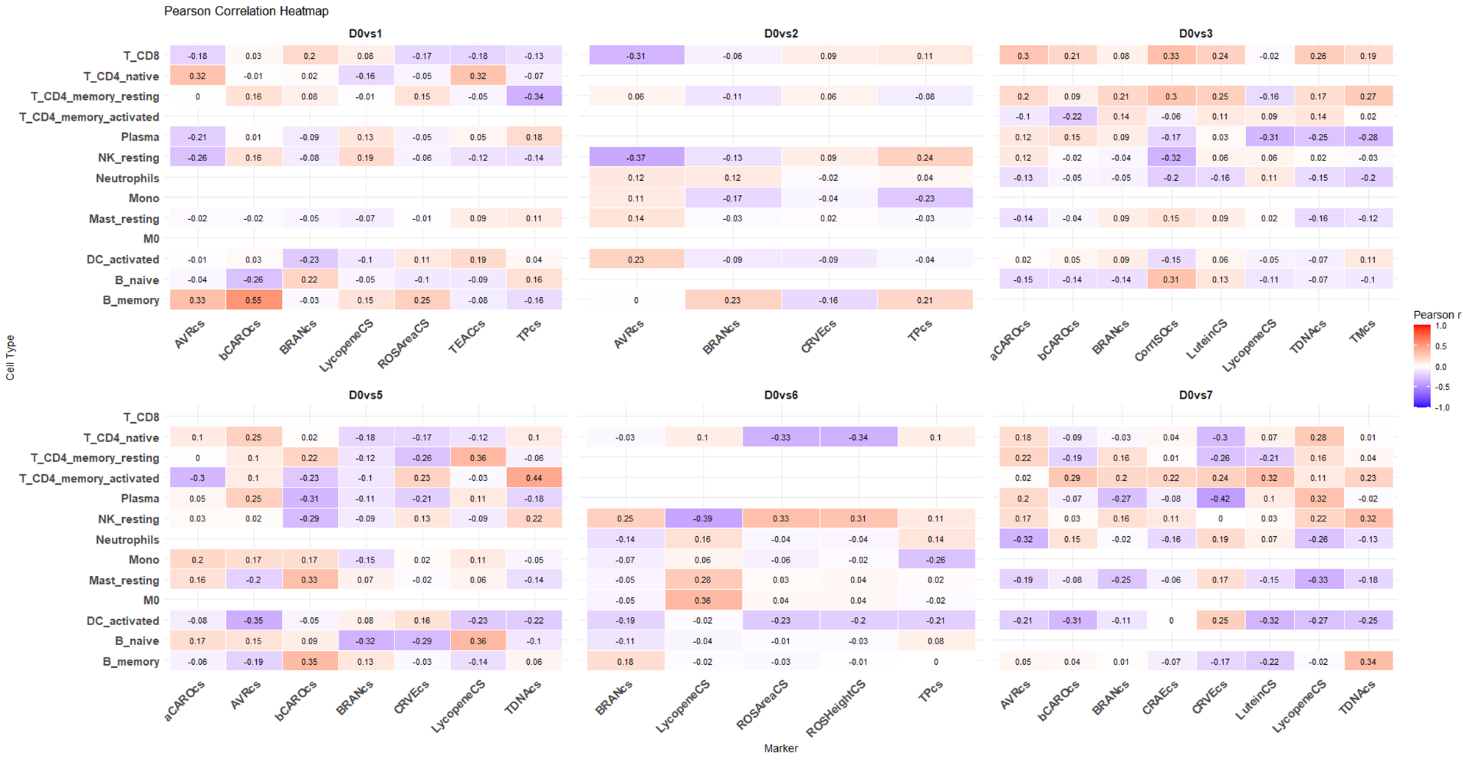


**Figure S3.** Heatmap of the Pearson correlation coefficients (R-values) between changes in cell proportions and alterations in oxidative stress markers, retinal microvasculature parameters, and circulating phytochemical levels following intervention.

**
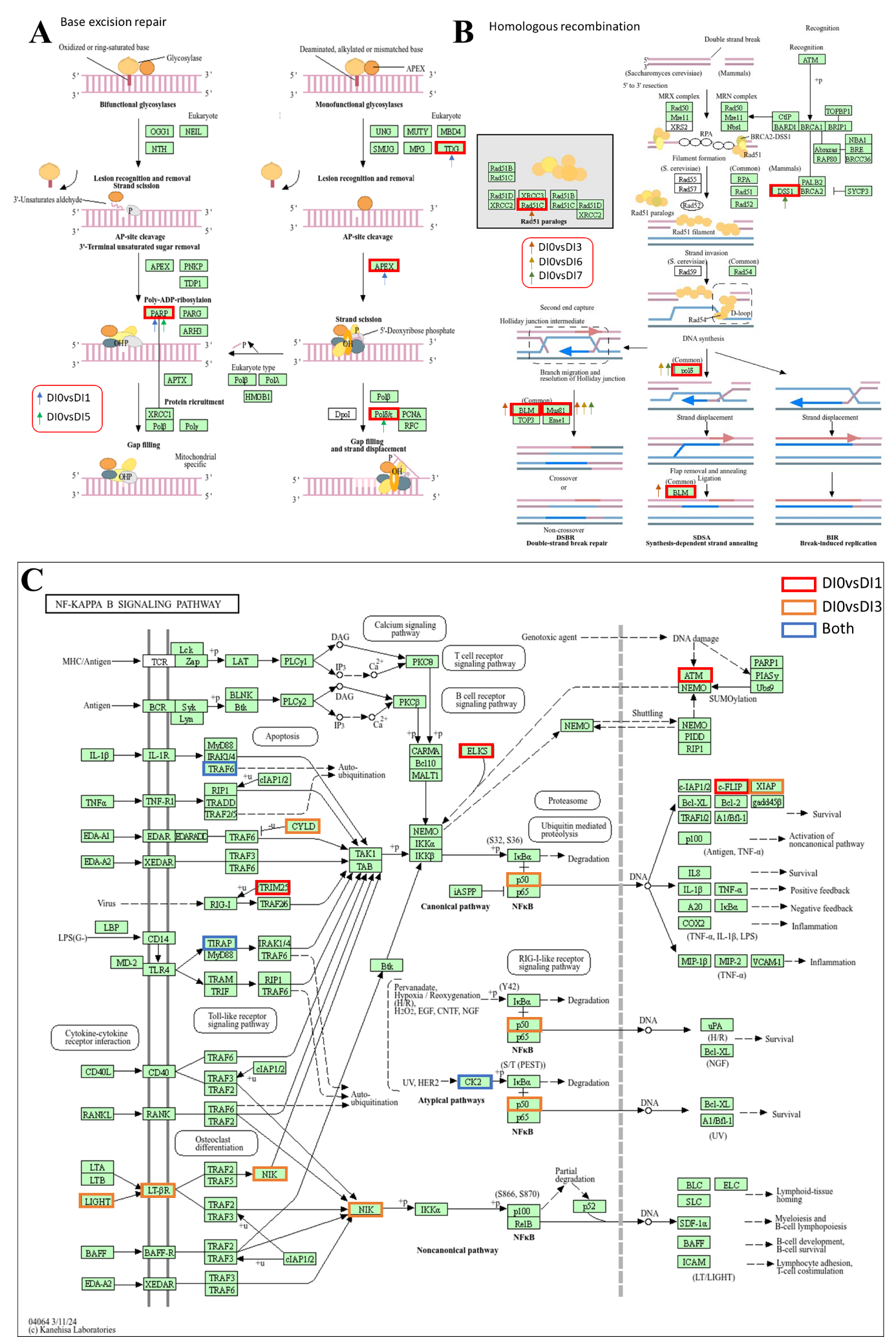
**

**Figure S4. Transcriptional modulation of DNA repair pathways in B cells and NF-KAPPA B signaling pathway in plasma cells.** A) Part of the upstream of base excision repair pathway influenced in B cells after intervention of blend 1 and 5 (DEGs are in the red box, the blue arrow represents blend 1, and the green represents blend 5). B) Homologous recombination pathway map from KEGG showing the regulation in B cell caused by blend 3, 6 and 7 (DEGs are in the red box, the orange arrow represents blend3, the yellow represents blend 6 and the green represents blend 7). C) KEGG metabolic pathway map shows significant DEG within the NF-KAPPA B signaling pathway in plasma cells after intervention. Genes in red frame are only regulated after intervention with blend 1, while the ones in orange frame are only regulated after intervention of blend 3, and the ones in blue are generated after intervention with both.


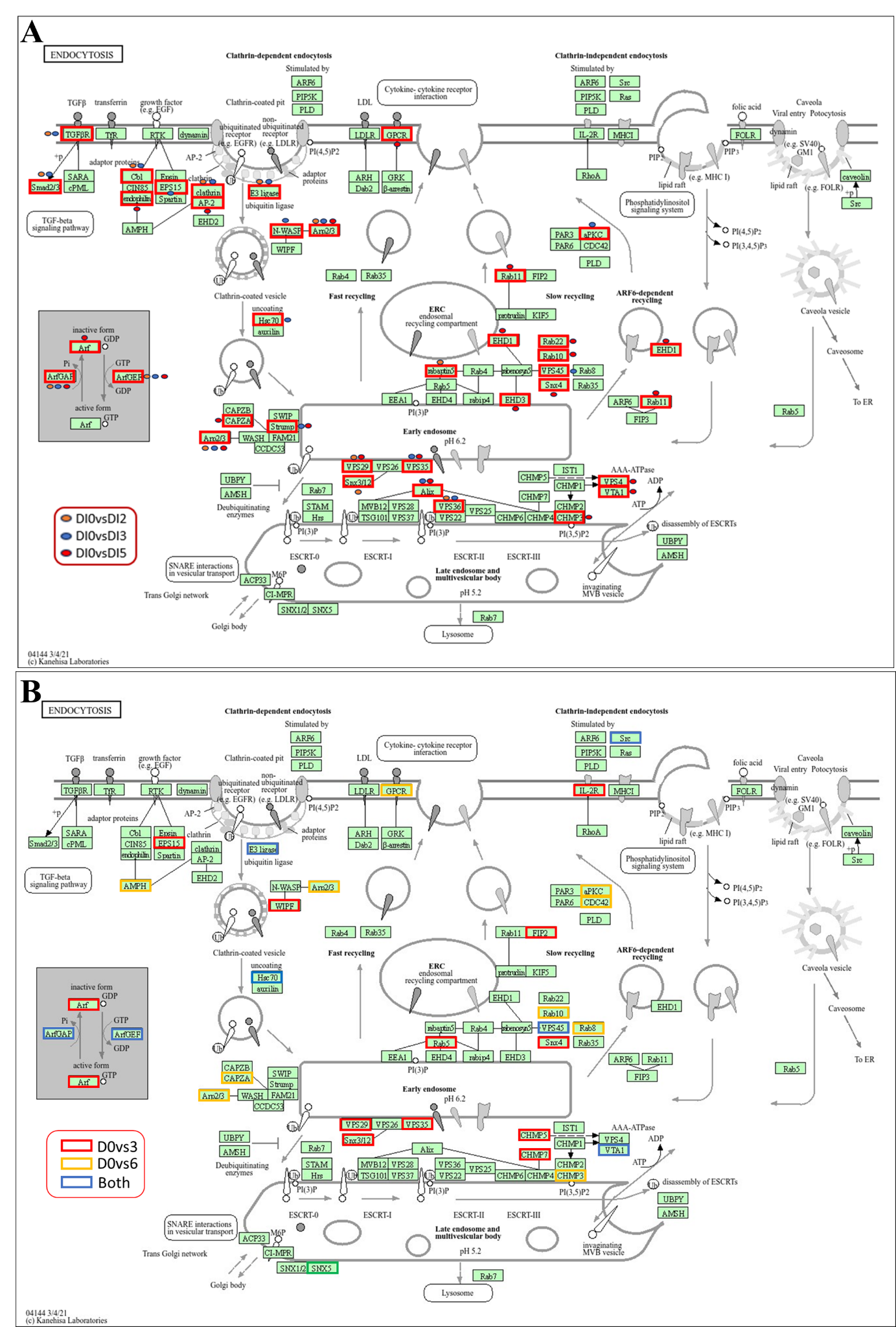


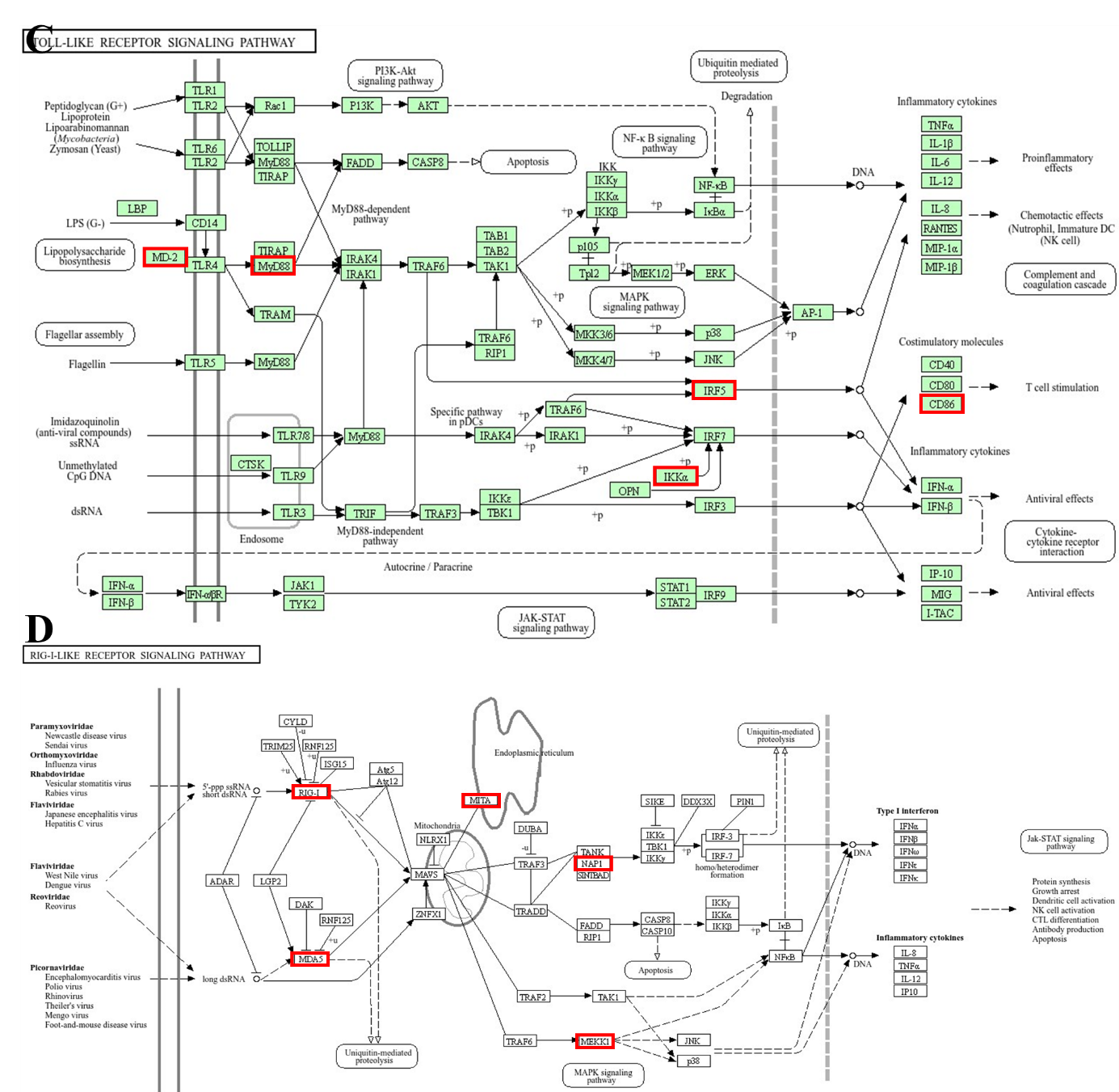


**Figure S5.** KEGG metabolic pathway maps showing DEGs within A) the endocytosis pathway in mast cells after intervention with different blends, where genes labeled with yellow ovals represent DEGs after blend 2, green ovals represent DEGs after blend 3, and red ovals represent DEGs after blend 5; B) the endocytosis pathway in dendritic cells (DCs) after intervention with blend 3 and blend 6; C) the TLR pathway in DCs after intervention with blend 5; D) the RIG-I signaling pathway in DCs after intervention with blend 1. For figures B-D, DEGs unique to blend 3 are highlighted in red frames, DEGs unique to blend 6 are highlighted in yellow frames, and DEGs common to both blends are highlighted in blue frames.


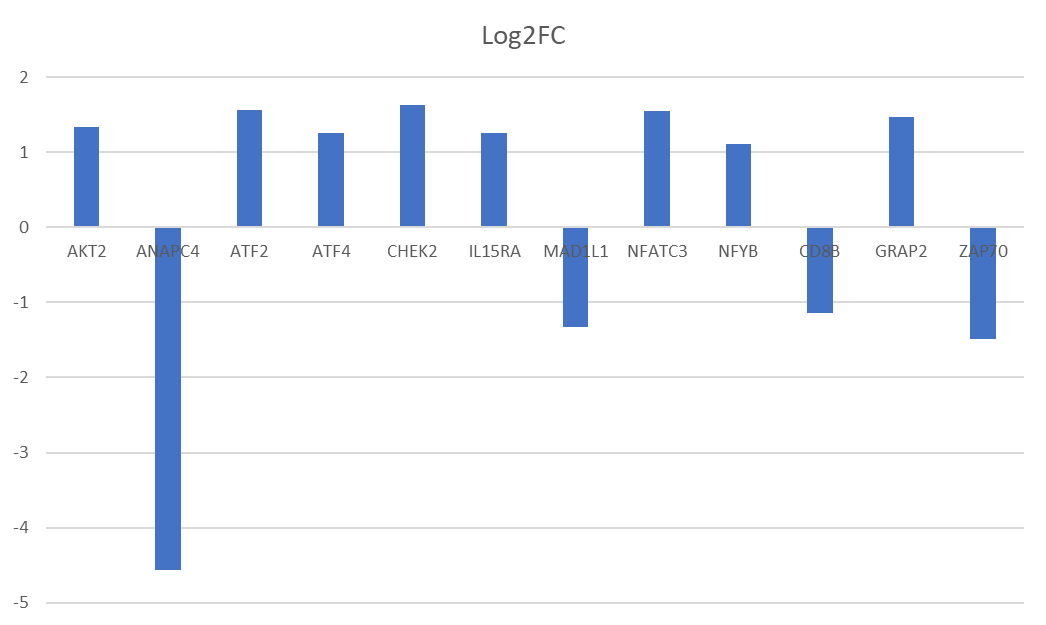


**Figure S6.** Log2FoldChange of DEGs in immune pathways in T cells after intervention with blend
